# The Long-Term Impact of COVID-19 on Disability after Post-Acute Rehabilitation: A Pilot Study

**DOI:** 10.3390/jcm13164694

**Published:** 2024-08-09

**Authors:** Claudia-Gabriela Potcovaru, Teodor Salmen, Ana Mădălina Potcovaru, Ioana-Miruna Săndulescu, Ovidiu Chiriac, Ana-Cristinel Balasa, Laura Sorina Diaconu, Daniela Poenaru, Anca Pantea Stoian, Delia Cinteza, Mihai Berteanu

**Affiliations:** 1 Doctoral School of Carol Davila, University of Medicine and Pharmacy, 050474 Bucharest, Romania; claudia-gabriela.potcovaru@drd.umfcd.ro (C.-G.P.);; 2 National Institute of Rehabilitation, Physical Medicine and Balneoclimatology, 030079 Bucharest, Romaniadaniela.poenaru@umfcd.ro (D.P.); 3 Faculty of Administration and Public Management, Bucharest University of Economic Studies, 010374 Bucharest, Romania; 4 Department of Gastroenterology and Internal Medicine, Carol Davila University of Medicine and Pharmacy, 050474 Bucharest, Romania; 5 Department of Diabetes, Nutrition and Metabolic Diseases, Carol Davila University of Medicine and Pharmacy, 050474 Bucharest, Romania; 6 Physical Medicine and Rehabilitation Department 9, University of Medicine and Pharmacy Carol Davila, 050474 Bucharest, Romania

**Keywords:** COVID-19, rehabilitation, disability, WHODAS 2.0, long-term management

## Abstract

**Background:** The long-term effect of the 2019 coronavirus (COVID-19) pandemic is not fully known. Severe cases of COVID-19 have resulted in disability that can be assessed in a biopsychosocial manner with the International Classification of Functioning, Disability and Health with the World Health Organization Disability Assessment Schedule 2.0 (WHODAS 2.0) questionnaire. This study aimed to evaluate the long-term effects on disability of COVID-19 three years after post-acute rehabilitation using WHODAS 2.0. **Methods**: This single-center cohort study included patients with severe COVID-19 who underwent immediate post-discharge post-acute rehabilitation intervention. Three years later, patients were assessed via telephone using the WHODAS 2.0 questionnaire. **Results:** Of the 69 patients identified in the hospital database, 27 responded. A total of 16 patients refused to respond due to emotional distress. The mean age was 63.4 ± 8.6 years, 81.5% were independent in the community, 55.3% had been previously admitted to the ICU, and the median rehabilitation hospitalization duration was 18 (11.5,24) days. Comorbidities included type 2 diabetes mellitus (DM) (55.5%), grade 2 high blood pressure (62.9%), pressure ulcers (37%), peripheral neurologic deficits (62.9%), and central neurological deficits (14.8%). ICU admission was significantly correlated with advanced rehabilitation needs (measured by the level of the rehabilitation (*p* < 0.01) and longer hospitalizations (measured by total days in the hospital (*p* < 0.001). The overall disability score was 35.09%, significantly influenced by DM and central neurological deficits. **Conclusions:** Central neurological deficits and DM are associated with higher disability scores. Tailored rehabilitation programs, ongoing medical assessment, integrated care models, and patient education are essential for improving long-term outcomes after COVID-19 disease.

## 1. Introduction

Coronavirus disease (COVID-19) is a disease caused by the severe acute respiratory syndrome coronavirus 2 (SARS-CoV-2), which spreads via airborne respiratory droplets [1].

The disease has multisystemic manifestations, affecting various organs and systems within the body, although the lungs are primarily affected in most cases [2]. In accordance with its clinical manifestation, the disease can be mild, severe, or critical, with a distribution of 81, 14 and, 5%, respectively [3]. The critical form is characterized by respiratory failure with a need for invasive mechanical ventilation (MV) [4], septic shock, and multiple organ dysfunction or failure [3]. The more severe the clinical form, the more evident the importance of comprehensive rehabilitation strategies to address the resultant disabilities and provide a supportive recovery for the patients.

The ongoing disability and long-term consequences following COVID-19 acute phase discharge remain hard to quantify [5]. Patients frequently encounter substantial physical and mental health issues post-discharge, including fatigue and impairments in respiratory, cardiac, renal, and neuropsychological functions. Some studies have revealed that these complications can last for at least six months and are often more significantly in those with severe cases of the disease [6,7]. In individuals with COVID-19, diabetes mellitus (DM) is known to be linked to a more severe manifestation of the illness, a more aggressive progression of the disease, and, subsequently, to an increased risk of death [8,9].

There are cases when the rehabilitation approach to COVID-19 necessitates the involvement of a full multidisciplinary team (MDT) [10], particularly for patients who needed prolonged admission in intensive care units (ICUs). This is due to their simultaneously development of pulmonary manifestations or of the complications of the disease itself alongside manifestations that are related to prolonged admission in an ICU ward, such as limb and respiratory muscle weakness, loss of physical energy, anxiety, depression, post-traumatic stress disorder (PTSD), or loss of cognitive functions [11]. The muscle catabolism increases and the muscle protein pool decreases during long ICU admission periods due to bed immobilization and a lack of physical activity (PA), known as ICU-acquired weakness (ICU-AW) [12]. It is considered that ICU-AW can last years after discharge, interfere with the quality of life, and determine an impaired functional status for the patients [13]. Moreover, the ICU patients without pre-existing neuromuscular disease who underwent MV for at least 7 days can develop ICU-acquired paresis (ICUAP), a factor that is associated with a longer need for MV. ICUAP is associated with both peripheral nerve and muscle involvement, and the term of peripheral neuropathy is understated, despite early studies that considered it to be the main effector of ICUAP. There is a significant association between corticosteroids and ICUAP, but it is not a necessary definite causal relationship, so the use of corticosteroids in critically ill patients should be restricted so that the benefits outsource the risks [14]. To minimize the sequels after ICU admission, the rehabilitation programs should start in the ICU wards. However, frequently, there is a lack of standardized protocols, staff, or time, and this contributes to an insufficient implementation of an early rehabilitation approach in the management of acute COVID-19 patients [15].

The rehabilitation of the patients after a critical form of COVID-19 should consist of an integrative approach and should be structured around the pulmonary rehabilitation of the restrictive pulmonary pathology, musculoskeletal and neurologic rehabilitation of critically ill patients that stayed in the ICU with ICU-AW and ICUAP, and the psychological rehabilitation of the associated cardiac pathology and psychological trauma. The post-acute phase of COVID-19 is characterized by patients who continued to experience symptoms of COVID-19 from 4 to 12 weeks after the onset. In this scenario, COVID-19 could have harmful consequences even after post-acute phase, depicting a new pathological condition: post-COVID-19 syndrome (PCS) or long COVID, representing a set of symptoms that persist for more than 12 weeks following COVID-19 and could not be accounted for by another diagnosis [16,17].

In general, the assessment of disability is challenging due to its context-dependent nature. Additionally, it has a subjective substrate, being influenced by its variable perception from the patient’s subjective experience along with the medical expert’s clinical observation [18]. The World Health Organization Disability Assessment Schedule (WHODAS 2.0) is a tool designed to measure disability in accordance with the International Classification of Functioning, Disability, and Health [19,20]. WHODAS 2.0 captures the level of functioning in six domains of activities in life: (1) cognition: understanding and communication (six items); (2) mobility: moving and getting around (five items); (3) self-care: hygiene, dressing, eating, and being alone (four items); (4) getting along: interacting with other people (five items); (5) life activities: domestic responsibilities, leisure, work, and school (eight items); (6) participation: joining in community activities (eight items). WHODAS 2.0 does not target a specific disease and can measure the impact of health or health-related interventions, such as rehabilitation interventions [21].

The long-term management of COVID-19 might be deficient because the exact consequences of this condition were not known. Studies are needed to report the effects of COVID-19 so that they can then be addressed in medical management strategies. WHO emphasizes the importance of innovative treatments in accordance with clinical evaluations. To obtain the best results for patients, the complete and personalized approach is promoted, which includes both clinical and paraclinical evaluations, but also drug treatments, such as corticotherapy, and oxygen supplementation [22].

The aim of this study is to assess the long-term disability outcomes in COVID-19 patients who underwent post-acute tertiary rehabilitation program based on the Start to Move As Soon As Possible (STM ASAP) protocol, three years post-discharge. The assessment of disability and health is conducted using the WHODAS 2.0 questionnaire. A second objective is to identify the factors related to the persistence of disability after discharge, and to evaluate the implications for the healthcare system, in order to be able to draw up policies that ease the management of post-COVID-19 patients’ cases and to be able to provide them with qualitative and long-term recovery and support.

## 2. Materials and Methods

### 2.1. Study Design, Protocol, and Patients

The present study was conducted in accordance with the principles of the Declaration of Helsinki and received approval from the Institutional Ethics Committee from the Romanian National Institute of Rehabilitation, Physical Medicine, and Balneology with the registration number NRCRT1/11.01.2021. In this single-center cohort study, patients were consecutively enrolled between May 2020 and September 2022.

Patients were diagnosed with COVID-19 by identifying the SARS-CoV-2 virus using either an antigen or PCR test. Patients included had a severe or critical form of COVID-19 according to the clinical manifestation and were treated in ICU units or other acute care units for COVID-19. After the vital risk was eliminated, they were referred immediate to a rehabilitation ward. Patient data were collected from hospital records after their discharged diagnostic according to the ICD-10 system of U07.1 (COVID-19, virus identified), and J96.9 (respiratory failure, unspecified). Data collected included phone numbers, demographic data (age, gender, education level, marital status), medical histories of pre-existing chronic disease, such as DM, hypertension, cardiovascular disease, respiratory conditions, ICU stay status, rehabilitation hospitalization duration, paraclinical data (capillary oxygen saturation at admission), and the clinical-based rehabilitation program initiation level that was set as we will mention below.

Inclusion criteria: patients with a positive diagnostic of a severe or critical form of COVID-19 who underwent immediate post-discharge tertiary rehabilitation therapy after their management for the acute phase in an ICU or other acute care unit for COVID-19.

Exclusion criteria were patients who refused to respond to the WHODAS 2.0 assessment, patients who died before being contacted, and patients with incomplete data in hospital records due to human error.

### 2.2. Assessment of Disability and Health Using the WHODAS 2.0 Questionnaire

From February to May 2024, all the patients identified were contacted via telephone and assessed for disability and health using the WHODAS 2.0 questionnaire. The 36-item version of WHODAS 2.0 was used for patients who continued to work or engage in school activities, while the 32-item (the 4 questions missing were related to work or school activities) version was used for those who were retired or unemployed, covering all 6 domains. The assessments were conducted by two experienced interviewers skilled in administering the questionnaire. The patients reported the level of difficulty they experienced when performing specific activities under normal conditions, choosing from the following options: “none”, “mild”, “moderate”, “severe”, and “extreme or cannot do”. Responses were scored as follows: “none” received a value of 1, and “extreme or cannot do” received a value of 5.

The following actions were performed to guarantee the reliability and validity of the data collected during telephone surveys using the WHODSA 2.0 questionnaire. Interviewers were trained to conduct the WHODAS 2.0 questionnaire according to a standardized protocol. They were also instructed to address any questions or misunderstandings that patients had during the survey. A second phone call follow-up interview with a subset of participants was conducted to confirm their responses.

### 2.3. Evaluation of Patients and Grouping Them into Levels for Starting the Rehabilitation Program

To establish the exact level of rehabilitation needed, patients were assessed based on the Start to Move ASAP (STM ASAP) protocol, adapting it subsequently after the patients evolved towards a better clinical–functional state [23].

After each assessment, the patients were grouped into six clinical–functional levels from 0 to 5; each level had a treatment option for physiotherapy and mobilization exercises that progressed in intensity and difficulty. A basic medical assessment (BMA) was made for each patient before deciding on their transfer. The BMA included four elements: cardiorespiratory evaluation (the patient was considered unstable if the mean blood pressure (BP) < 60 mmHg or >120 mmHg, FiO_2_ > 60%, PaO_2_/FiO_2_ < 200, or respiratory rate (RR) > 30 bpm); neurological evaluation (the patient was unstable while having a high intracranial pressure—ICP > 20 mmHg or cerebral perfusion pressure > 70 mmHg); the presence of acute surgery; and the body temperature > 40 °C. When one of the four primary elements was present, the patient was ranked as level 0 and failed the test. The patients were transferred to a rehabilitation unit only if they had passed the BMA.

Furthermore, the patient’s ability to cooperate was assessed using the Standardized Five Questions (S5Q). Each correct response was scored with 1. A non-cooperative patient has an S5Q score ˂ 3, and a responsive and adequate patient has a SQ5 score ≥ 3 [24,25].

Muscle strength has been assessed using the Medical Research Council (MRC) sum-score for three muscle groups of the upper and lower limbs bilaterally (arm abductors, forearm flexors, hand extensors, hip flexors, knee extensors, and foot dorsiflexors). Each muscle group receive a score between 0–5, with a maximum possible score of 60 points, which were linked with a normal muscle function. Here, 0 represents no visible contraction; 1 represents visible muscle contraction but no limb movement; 2 represents active movement but not against gravity; 4 represents active movement against gravity and resistance; and 5 represents active movement against full resistance. An MRC sum score below 36 indicates significant weakness and corresponds to an incapacity of the muscle to perform against resistance [26]. To ensure accuracy and reliability, the doctors who evaluated the patients were trained to maintain consistency when assessing muscle strength using the MRC sum score. At least two trained doctors independently evaluated the muscle strengths of each patient and then reached a consensus as to the final score.

The ability to maintain balance was assessed using a simplified version of the Berg balance scale (BBS) consisting of 3 of the 14 items from the BBS that were relevant for the initial evaluation of patients with long admission durations in the ICU. Each of the items are scored from 0 to 4, and the items included were, respectively, sitting to standing (BBS sit to stand), standing unsupported (BBS standing), and sitting with the back unsupported but the feet supported on the floor or on a stool (BBS sitting) [27].

Using BMA, S5Q, and MRC sum score, as well as the modified BBS, the patients were included in the 5 clinical–functional levels, respectively:

- Level 0 includes the patients that did not pass the BMA assessment, who were still admitted into ICU units, and who have an S5Q of 0; the rehabilitation program for them includes 2 h turning, splinting, and positioning.
- Level 1 includes the patients that passed the BMA, with an S5Q score between 1 to 5, for whom transfer to a chair is not allowed because of a neurological condition; the rehabilitation program for them includes 2 h turning, splinting, Fowler′s position, a passive/active range of motion (ROM), passive/active leg and/or arm cycling in bed, neuromuscular electrical stimulation (NMES), and activities of daily living (ADL) performance.
- Level 2 includes patients that passed the BMA with an S5Q score ranging between 3 to 5, for whom transfer to a chair is allowed but the patient cannot do it without the help of another person; the rehabilitation program includes 2 h turning, splinting, upright siting position in bed, passive transfer from a bed to a chair, a passive/active ROM, passive/active leg and/or arm cycling in bed, NMES, and ADL performance.
- Level 3 includes patients that passed the BMA with a minimum S5Q score of 4, and an MRC sum score ≥ 36, with BBS sit to stand = 0, BBS standing = 0, and BBS sitting ≥ 1; the rehabilitation program includes 2 h turning, passive transfer from a bed to a chair, sitting out of bed, standing with at least 2 persons assisting, a passive/active ROM, resistance training for arms and legs, active leg and/or arm cycling in a bed or chair, standing (with assistance/frame), NMES, and ADL performance.
- Level 4 includes patients that passed the BMA with an S5Q score of 5 and an MRC sum score ≥ 48, with BBS sit to stand ≥ 0, BBS standing ≥ 0, and BBS sitting ≥ 2; the rehabilitation program includes active transfers from a bed to a chair, sitting out of bed, standing with 1 person assisting, a passive/active ROM, resistance training for arms and legs, active leg and/or arm cycling in a bed or chair, walking (with assistance/frame), NMES, and ADL performance.
- Level 5 includes patients that passed the BMA with an S5Q score of 5 and an MRC sum score ≥ 48, with BBS sit to stand ≥ 1, BBS standing ≥ 2, and BBS sitting ≥ 3; the rehabilitation program includes active transfers from a bed to a chair, sitting out of bed, standing, passive/active ROM, resistance training for arms and legs, active leg and/or arm cycling in bed or chair, walking (with assistance), NMES, and ADL performance.

The patients that had at least clinical–functional level 3 were assessed further using the Timed Up and Go test (TUG), ten-meter walk test (10MWT), four-meter gait speed Test (4MGS), and six-minute walk distance (6MWD), along with a modified Borg Rating of Perceived Exertion Scale (RPE) [28,29,30,31].

The metabolic equivalent of task (MET) value was estimated depending on the PA that the patient could achieve. Conventionally, 1 MET represents an oxygen uptake of 3.5 milliliters/kilogram of body weight/minute and is considered the energy cost of a person while siting and rest. MET values were estimated using a chart that provides approximate MET values for a variety of light, moderate, and vigorous activities. This chart helped quantify the energy expenditure associated with each activity performed by the patients during their rehabilitation sessions. MET values were used in the analysis to assess the overall PA level. Light-intensity PA is defined as an MET between 1.1 and 2.9, moderate-intensity PA is defined as an MET between 3.0 and 6.9, and vigorous PA is defined as 6 METs or more [32].

### 2.4. Statistical Analysis

The data were organized using Excel Office 365, Version 16.0 for Windows 10 and Gnu PSPP 1.4.1 software, which facilitated a thorough statistical assessment for the gathered parameters. The results are presented as the mean and standard deviation (SD) for normally distributed data or median and interquartile range (IQR), for non-normally distributed data. In univariant correlation analysis, Pearson or Spearman coefficients were used depending on the distribution of the variables. Chi-square tests were employed to examine the associations between categorical variables. The distribution of the data was tested using the Kolmogorov–Smirnov test. The scoring of WHODAS 2.0 was performed using the complex scoring method available from the WHO website [33].

## 3. Results

**Figure 1:**
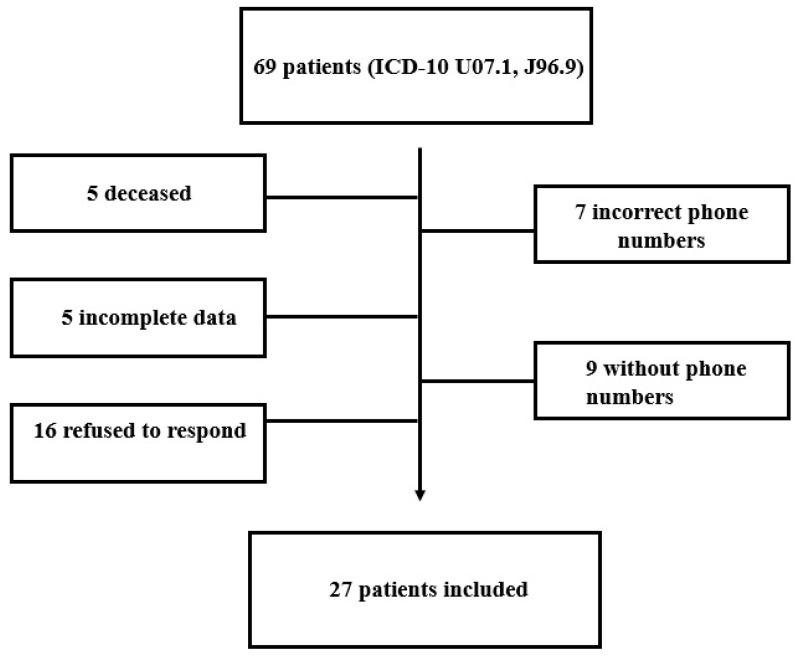
Patient inclusion flowchart.

A total of 69 patients were found in hospital database with the inclusion criteria mentioned in Section 2. All patients were contacted via telephone; of these, 5 patients were deceased (information from caregivers), 7 had incorrect contact information, 9 lacked phone numbers, 5 had incomplete medical records, and 16 declined to participate in the questionnaire evaluation of disability level. Ultimately, 27 eligible patients had their disability evaluated by the WHODAS 2.0 questionnaire and were included in the final analysis, as illustrated in Figure 1.

The patients’ demographic characteristics are summarized in Table 1. Our cohort gender distribution is balanced, with 51.9% females and 48.1% males, with no statistically significant difference between genders. The mean age of the analyzed patient sample was 63.4 ± 8.6 years. Among the patients, 81.5% were independent in the community, while 18.5% required social assistance. The mean number of years in education (school, college, and university) for patients was 13.14 ± 2.9 years. In terms of marital status, 70.4% were married, 14.8% were widowed, and 11.1% were divorced. Regarding employment status, 77.8% were retirees, while 22.2% were employed.

A total of 59.3% of patients were admitted to the ICU. The average hospitalization duration in rehabilitation unit was 19.14 ± 8.06 days. The median peripheral oxygen saturation at admission was 90% (88.5 to 91.5), and the median oxygen therapy requirement was 1.24 L/min (IQR of 1 to 3), as seen in Table 2. The rehabilitation program initiation levels are also presented in Table 2, with 22.63% at level 2, 22.63% at level 3, 22.22% at level 4, and 18.52% at level 5.

Table 3 summarizes patients’ comorbidities, where 62.9% had Grade 2 high BP (HBP), 18.5% had Grade 1 HBP, and 55.5% had type 2 DM. Additionally, the present infections were 74.1% urinary tract infections and 22.2% Clostridium difficile. Furthermore, 18.5% had Grade 2 pressure ulcers, 14.8% had Grade 1 pressure ulcers, and 3.7% had Grade 3 pressure ulcers. Other notable conditions included pneumothorax in one patient, peripheral neurological deficits in 62, 9.2% of patients, and central neurological deficits in 14.8% of patients.

Regarding the WHODAS 2.0 questionnaire, the overall disability score was 35.09%, with the distribution in domains shown in Table 4.

The disability scores for specific domains were as follows: 22.99% in the understanding and communicating domain, 35.92% in the getting around domain, 28.00% in the self-care domain, 28.89% in the getting along with people domain, 51.79% for household chores, 3.13% for work-related domains (based on the six patients who completed this section), and 39.81% in the participation in society domain.

### 3.1. ICU Admission History

Regarding ICU admission history, in terms of the rehabilitation admission status, there was statistical significance only for level 2 and level 5 of the rehabilitation admission levels, i.e., at *p* = 0.005 and, respectively, *p* = 0.048. No association was observed regarding paraclinical parameters, for the O_2_ saturation level at admission of *p* = 0.204, nor for comorbidities, namely DM and HBP, at *p* = 0.381 and *p* = 0.132, respectively.

### 3.2. Rehabilitation Admission Levels

DM, HBP, and both central neurologic deficits were not associated with the rehabilitation admission levels, while for the peripheral neurologic deficit, only level 5 had a significant association of *p* = 0.028, while regarding the number of hospitalization days required during the rehabilitation management approach, the significant correlation was positive and only present for level 2 (r = 0.582, *p* = 0.001).

### 3.3. Rehabilitation Hospitalization Duration

There were negative correlations between the rehabilitation hospitalization duration and the disability scores in the six WHODAS 2.0 domains, but these correlations are not statistically significant.

No significant association between DM, HBP, and central or peripheral neurologic deficit was obtained, with *p* = 0.246, and, respectively, *p* = 0.08.

Central neurologic deficits are positively correlated with a higher disability score in all six domains of WHODAS 2.0. (*p* < 0.01) and with longer hospital stays (r = 0.556, *p* < 0.01). ICU admission is negatively correlated with most of the six WHODAS 2.0 domains as follows: r = −0.397, *p* = 0.041 for the cognition domain, r = −0.579, *p* = 0.002 for the mobility domain, r = −0.455, *p* = 0.017 for the self-care domain, r = −0.307, *p* = 0.119 for the getting along with people domain, r = −0.433, *p* = 0.024 for the life activities domain, and r = −0.42, *p* = 0.029 for the participation in society domain.

A longer hospital stay in rehabilitation unit is linked with ICU admission (r = 0.399, *p* < 0.05).

### 3.4. WHODAS 2.0 Domains and Their Correlations

Cognition disability scores (domain 1) from the WHODAS 2.0 questionnaire are positively correlated with central neurologic deficits (r = 0.562, *p* = 0.002) and negatively correlated with ICU admission history (r = −0.397, *p* = 0.041). No significant correlation was observed with length of hospital stays, oxygen saturation, DM, peripheral neurologic deficits, or HBP, as seen in Table 5.

Mobility disability scores (domain 2) from WHODAS 2.0 are positively correlated with DM (r = 0.383, *p* = 0.048) and central neurologic deficits (r = 0.495, *p* = 0.009), both being significant, and negatively correlated with ICU admission history (r = −0.579, *p* = 0.002). No significant correlation with hospitalization duration, oxygen saturation, peripheral neurologic deficits, or HBP was found, as shown in Table 5.

Self-care disability scores (domain 3) from WHODAS 2.0 are positively correlated with central neurologic deficits (r = 0.683, *p* = 0.000) and negatively correlated with ICU admission history (r = −0.455, *p* = 0.017). No significant correlation with hospitalization duration, oxygen saturation, DM, peripheral neurologic deficits, or HBP was found, as shown in Table 5.

Getting along with people (domain 4) from WHODAS 2.0 disability scores are positively correlated with central neurologic deficits (r = 0.550, *p* = 0.003) but not significantly correlated with ICU admission history, length of hospitalization duration, oxygen saturation, DM, peripheral neurologic deficits, or HBP was found, as shown in Table 5.

Life activities (disability domain 5) from WHODAS 2.0 scores are positively correlated with oxygen saturation and central neurologic deficits (r = 0.405, *p* = 0.036) and negatively correlated with ICU admission history (r = −0.433, *p* = 0.024). No significant correlation with length of hospitalization duration, DM, peripheral neurologic deficits, or HBP was found, as shown in Table 5.

Participation in society (disability domain 6) from WHODAS 2.0 scores are positively correlated with central neurologic deficits (r = 0.599, *p* = 0.001) and negatively correlated with ICU admission history (r = −0.420, *p* = 0.029). No significant correlation with length of hospitalization duration, oxygen saturation, DM, peripheral neurologic deficits, or HBP was found, as shown in Table 5.

## 4. Discussion

The global COVID-19 pandemic has profoundly modified our lifestyle, including work, social interactions, and travel patterns. It is also associated with a significant pressure on the healthcare system. To thoroughly understand the pandemic’s effects on patients, disability research must be conducted across a long time span to increase the overall reaction perception, to determine the long-term effects on the general population’s health, and to develop adequate planning for providing the necessary resources and care where it is most needed. Moreover, limited attention was paid to the disabilities encountered by the patients affected by COVID-19, especially for the ones that required post-acute rehabilitation therapy after discharge from the acute phase of the disease. The reported data about disability are scarce, and while some studies report data at short follow-up intervals, such as one or six months, there is a lack of consistent research addressing functional impairment over longer and more relevant time spans [34,35].

The completion rate of the questionnaires is not so high for several subsequent reasons. For example, the refusal to answer the questionnaire might be because COVID-19 was too painful to remember, a reason that aligns with other studies that reported that many survivors experienced significant emotional distress, including fear, anxiety, and loneliness, during and after discharge, particularly following ICU hospitalization [36]. After being admitted to the ICU, 25% to 33% of survivors may develop PTSD, which can last for up to five years [37]. Stressors, like those from the present COVID-19 pandemic, were experienced by survivors of the 2002–2003 SARS outbreak, and included high media attention, societal stigma, worries about infecting loved ones, the loss of close family members, and feelings of survivor’s guilt. These psychological difficulties highlight some of the crucial factors that should be considered while analyzing the long-term psychological effects of COVID-19.

Overall disability in our cohort was 35.9% three years after COVID-19, as assessed by the WHODAS 2.0 questionnaire. On this scale, 0 represents no disability and 100% represents complete disability, while a score of 35.9% indicates moderate difficulty in performing certain tasks. In similar studies comparing WHODAS 2.0 with other questionnaires, such as the EuroQol Five-Dimensional Five-Level (EQ-5D-5L), WHODAS 2.0 often reports higher values, reflecting more severe sequelae. This may be because WHODAS 2.0 focuses on specific functional difficulties, whereas EQ-5D-5L assesses overall feelings about health. Therefore, for a thorough assessment of a patient’s health-related disability and functional status, it is important to assess it with multiple questionnaires simultaneously [38].

Higher rates of disability were linked to ICU admission history, especially for admissions rehabilitation level 2 and level 5, because for levels 3 and 4, no significant association was found [39]. Rehabilitation level 2 included patients that cannot transfer from a bed to a chair without help. The rehabilitation procedures for this category included turning at 2 h, splinting, upright sitting position in bed, passive transfer from bed to chair, a passive/active ROM, NMES, and ADL performance. This level indicates that the severity of the initial condition is influenced by ICU admission history and requires extensive rehabilitation support.

The relationship between ICU admission history and various disability domains as measured by the WHODAS 2.0 questionnaire provides valuable insights into the impact of critical care on long-term functional outcomes. The correlations observed in the study suggest that ICU admissions are associated with specific patterns of disability across different functional domains. These findings highlight the nuanced relationship between ICU admissions and long-term disabilities across different domains. Central neurologic deficits consistently show a strong positive correlation with higher disability scores, indicating a significant impact on various functional domains. In contrast, ICU admissions tend to correlate negatively with disability scores in most domains, suggesting possible rehabilitation benefits or the effectiveness of post-ICU care in improving functional outcomes.

Other studies, based on ICF domains, used the Barthel index (BI), Functional Independence Measure (FIM), and SF-36 Health Questionnaire, and reported disability after 24 sessions, each lasting 45 min, using a robotic hand exoskeleton. They showed significant improvement in specific areas, like self-care and transfers, but not in overall scores or mobility for BI. The SF-36 highlighted improvements in various health-related quality-of-life domains, but not in the physical functioning role (t = −1.8680, *p* = 0.0763). The specific improvements were as follows: bodily pain (t = −3.6703, *p* = 0.0022), general health (t = −2.3381, *p* = 0.0281), mental health (t = −3.3018, *p* = 0.0030), physical functioning (t = −2.2575, *p* = 0.0339), emotional role (t = −2.6363, *p* = 0.0161), social functioning (t = −3.1250, *p* = 0.0049), and vitality (t = −3.0302, *p* = 0.0059) [40].

Diabetic myopathy, characterized by muscle weakness, is a complication of DM that needs to be addressed in rehabilitation programs because it leads to exercise intolerance and a diminished quality of life. Also, sarcopenia is a common condition in the ICU-treated patients, reaching a 68% incidence, and needs to be taken into consideration as an important element in the critically ill patient’s rehabilitation process. It leads to longer hospital stays and a lower body mass index and is associated with older age (68 as compared to 55 years, *p* < 0.001) [41]. According to other studies, peripheral muscle strength declines by about 20% every week that patients stay in the hospital bed [42]. A multidisciplinary approach is mandatory in the management of these patients to improve their recovery process. Consequently, the rehabilitation program combines conventional therapy with modern rehabilitation techniques, including robotic training. Along with exercise, dietary advice from a diabetologist is also included to maximize patient recovery. These individuals need to consume more protein and nutritional supplements, which contribute to the comprehensive care required for such complex cases [43,44]. Nutritional advice is essential not only for managing sarcopenia but also for addressing pressure ulcers. In our study, 37% of patients had pressure ulcers of various grades, and malnutrition is known to delay healing. Increasing energy and protein intake, along with supplementation of zinc, vitamin A, and vitamin C, can support the healing process. Furthermore, ensuring that patients’ drinking water is enhanced with micronutrients, like calcium, magnesium, and potassium, might help promote recovery [45,46]. Furthermore, there is a connection between severe COVID-19 outcomes and malnutrition via both direct and indirect pathways. By lowering lymphocyte numbers, impairing phagocytic function, and decreasing cellular immunity and physical barriers, immunological protection is decreased. Additionally, malnutrition worsens the inflammatory response [47,48].

Factors increasing the risk of severe COVID-19 include an older age, male gender, sedentary lifestyle, and pre-existing health conditions, such as asthma, various chronic diseases (including kidney, lung, and liver diseases), DM, cardiovascular conditions, mental health disorders, smoking, sickle cell disease, and the use of corticosteroids or immunosuppressive medications [49,50,51,52,53]. In our findings, DM significantly impacts certain disability domains, such as mobility, and is associated with higher rates of peripheral neurologic deficits. This aligns with the known risk of DM in severe COVID-19 and suggests that the impact extends into the recovery phase, potentially complicating rehabilitation efforts [54]. Additionally, patients with DM experience a slower rehabilitation process. Rehabilitation programs should include multimodal interventions, such as physical exercise, nutritional changes, behavioral therapy, and cognitive approaches [55]. In our study, HBP does not show a direct significant relationship with disability scores or rehabilitation admission levels, most probably due to the small sample size, despite HBP generally being considered a risk factor for severe COVID-19. The elevated BP levels may be influenced by dexamethasone, which was included in the management of COVID-19. Dexamethasone can cause sodium retention, vasoconstriction, and sympathetic nerve activation [56].

### Healthcare Management Implications

Some healthcare strategies can be taken into consideration to improve the long-term management of patients following COVID-19 and to reduce disability levels, and, consequently, reduce the healthcare burden. For this objectives, four main pillars should be followed. (1) The rehabilitation program must be personalized: There is a need to implement individualized rehabilitation programs and rehabilitation cycles that address specific functional deficits and adapt to the changing needs of patients over time. These strategies can be modified using goal attainment scaling (GAS) [57]. GAS should be SMART (specific, measurable, achievable, relevant, and time-bound). (2) Continuous medical evaluation: Regular follow-ups are needed to track progress and make necessary adjustments to rehabilitation strategies (3) Integrated medical models: A multidisciplinary approach that includes physical, cognitive, and psychological support is needed to address the diverse impact of long-term COVID-19 disability. (4) Patient Education: Patients and caregivers need constant education and support to enhance adherence to rehabilitation protocols and self-management practice.

The limitations of the present study are as follows: it used a small sample size, which could reduce the statistical power and adaptability of our findings. Additionally, due to patients’ refusal to participate in the study for several reasons (ex. PTSD) or due to their deaths, the dataset may be biased, as the characteristics of those who participated in the follow-up may differ from those who did not; assessing health-related disability using only one questionnaire may limit the comprehensiveness of the disability evaluation; assessment of disability by WHODAS 2.0 questionnaire was conducted only at the follow-up phone call, after a period during which patients were not reevaluated for other potential disability generator conditions; from the patient’s perspective, a main limitation is the insufficient referral to rehabilitation services of the ICU-treated patients. Taking this into consideration, future studies should aim to include larger sample sizes, utilize more comprehensive assessment tools, and ensure that patients are called to come into the hospital to carry out a clinical evaluation and face-to-face tests.

## 5. Conclusions

Three years after the COVID-19 pandemic, the overall disability score measured by the WHODAS 2.0 questionnaire reflects a moderate difficulty in performing certain tasks. A history of ICU admission is significantly associated with more advanced rehabilitation techniques, as indicated by higher rehabilitation levels at admission, and also with a need for a longer hospitalization in rehabilitation units. In all six domains of WHODAS 2.0, the central neurological deficit was correlated with higher scores and with a higher degree of disability. In contrast, ICU admission was negatively correlated with most WHODAS 2.0 domains, which may suggest the possible benefits of rehabilitation programs or the effectiveness of post-ICU care. DM has a significant impact on certain domains of disability, particularly mobility, and is associated with higher rates of peripheral neurological deficits. The study also highlights the need for tailored and personalized rehabilitation programs, ongoing medical assessment, integrated medical models, and patient education to improve long-term management after COVID-19, in order to reduce disability levels. The impact of the COVID-19 pandemic on disability in patients extends into the recovery phase, potentially complicating rehabilitation efforts, especially for patients with pre-existing conditions, such as DM.

## Figures and Tables

**Table 1:** Patients’ demographic characteristics.

Characteristics	*n* = 27
Age (years), mean (SD)	63.4 ± 8.6
Females, %, Males, %	51.9% 48.1%
Independent in the community, %	81.5%
Education (years), mean (SD)	13.14 ± 2.9 years
Marital status (married), %	70.4%
Employment status (retirees), %	77.8%

SD—standard deviation.

**Table 2:** Patients’ admission characteristics.

Characteristics	*n* = 27
ICU unit admission (%)	59.3%
Number of hospitalization days, median (IQR)	18 (11.5,24)
Rehabilitation level at admission	
Level 2 (%)	29.63%
Level 3 (%)	29.63%
Level 4 (%)	22.22%
Level 5 (%)	18.52%
Peripheral oxygen saturation (%), median (IQR)	90% (88.5 to 91.5)
Average oxygen therapy requirement (L/min), median (IQR)	1.24 L/min (1 to 3)

ICU—intensive care unit; IQR—interquartile range.

**Table 3:** Patients’ comorbidities.

Characteristics	*n* = 27
Type 2 Diabetes Mellitus (%)	55.5%
Grade 1 HBP (%)	18.5%
Grade 2 HBP (%)	62.9%
Urinary tract infection (%)	74.1%
Clostridium difficile infection (%)	22.2%
Pressure ulcers grade I (%)	14.8%
Pressure ulcers grade II (%)	18.5%
Pressure ulcers grade III (%)	3.7%
Peripheral neurological deficit	62.9%
Central neurological deficit	14.8%

HBP—high blood pressure; ICU—intensive care unit.

**Table 4:** WHODAS 2.0 scores across six categories.

Disability Categories		Disability Percentages
Cognition		22.99%
Mobility		35.93%
Self-care		28.01%
Getting along with people		28.89%
Life activities	Household	51.79%
	Work or school activities	3.13%
Participation in society		39.81%
Overall disability		35.09%

**Table 5:** Statistical Correlations and Significance for WHODAS 2.0 Domains and Clinical Metrics.

	Days in Hospital	O_2_ Saturation	DM	Peripheral Neurologic Deficit	Central Neurologic Deficit	HBP	ICU Admission
Cognition (d1)	r = −0.245, *p* = 0.217	r = 0.308, *p* = 0.119	r = 0.280, *p* = 0.497	r = −0.151, *p* = 0.453	r = 0.562, *p* = 0.002	r = −0.082, *p* = 0.658	r = −0.397, *p* = 0.041
Mobility (d2)	r = −0.251, *p* = 0.207	r = 0.261, *p* = 0.188	r = 0.383, *p* = 0.048	r = −0.158, *p* = 0.430	r = 0.495, *p* = 0.009	r = 0.028, *p* = 0.888	r = −0.579, *p* = 0.002
Self-care (d3)	r = −0.275, *p* = 0.165	r = −0.293, *p* = 0.138	r = −0.263, *p* = 0.184	r = −0.204, *p* = 0.308	r = 0.683, *p* = 0.000	r = −0.093, *p* = 0.646	r = −0.455, *p* = 0.017
Getting along with people (d4)	r = −0.206, *p* = 0.302	r = 0.251, *p* = 0.206	r = 0.308, *p* = 0.119	r = −0.190, *p* = 0.342	r = 0.550, *p* = 0.003	r = 0.049, *p* = 0.810	r = −0.307, *p* = 0.119
Life activities (d5)	r = −0.306, *p* = 0.121	r = −0.405, *p* = 0.036	r = 0.237, *p* = 0.234	r = −0.114, *p* = 0.572	r = 0.505, *p* = 0.007	r = 0.157 *p* = 0.433	r = −0.433, *p* = 0.024
Participation in society (d6)	r = −0.214, *p* = 0.283	r = 0.322, *p* = 0.101	r = 0.275, *p* = 0.164	r = −0199, *p* = 0.321	r = 0.599, *p* = 0.001	r = 0.104, *p* = 0.604	r = −0.420, *p* = 0.029

DM—diabetes mellitus; HBP—high blood pressure; ICU—intensive care unit.

## Data Availability

Data provide upon request.
